# Bibliometric Insights Into the Infodemic: Global Research Trends and Policy Responses: Quantitative Research

**DOI:** 10.2196/76378

**Published:** 2025-10-14

**Authors:** Sijia Wang, Linan Zhang, Yang Liu, Xin Feng, Shipeng Ren

**Affiliations:** 1 Department of Urban Construction, Hebei Normal University of Science and Technology, Qinhuangdao, China; 2 School of Marxism, Shijiazhuang Tiedao University, Shijiazhuang, China; 3 School of Arts and Communication, Hebei University of Engincering Science, No.11, Gongbei Road, Qiaoxi District, Shijiazhuang, Hebei, 050018, China, 86 17732192671; 4 The Institute for Social and Cultural Research, Macau University of Science and Technology, Macau, China; 5 School of Economics and Finance, Xi'an Jiaotong University, Xi'an, China

**Keywords:** infodemic, bibliometrics, visualization, information spreading, social media, information governance

## Abstract

**Background:**

Amidst the COVID-19 pandemic, the proliferation of misinformation on social media, termed the “infodemic,” has complicated global health responses.

**Objective:**

This study aims to identify research trends and information-making in the context of this challenge. This paper synthesizes key areas of scholarly investigation into the COVID-19 infodemic, both within China and internationally, to guide public health strategies and the management of public sentiment.

**Methods:**

By employing a bibliometric approach, using CiteSpace software, we conducted a visual analysis of the global literature, covering a total of 1437 publications from the Web of Science and the China National Knowledge Infrastructure core databases between 2016 and 2025, focusing on publication trends, citation frequencies, and keyword clusters.

**Results:**

After analysis, the results reveal distinct focal points in the research priorities of Chinese and international scholars. International studies often focus on machine learning and public psychology, while Chinese research tends to address information control and safeguarding. Common ground is found in the interest in preventing the spread of misinformation. While literature on COVID-19 abounds, cross-national systematic reviews are limited.

**Conclusions:**

This paper fills this gap through a comparative bibliometric analysis, offering valuable insights for information management, media communication, and public administration, thus charting new directions for future research.

## Introduction

The COVID-19 pandemic has precipitated unprecedented disruptions across global business, economic, and cultural landscapes. Simultaneously, the “infodemic” of misinformation has emerged as a formidable adversary, undermining efforts to restore social stability. Misinformation, typically characterized by the dissemination of unverified content via informal networks, lacks empirical substantiation and rational debate on matters of public importance [1,2]. The pandemic has expedited the spread of such misinformation, leading to widespread information anxiety and eroding public trust [3]. The infodemic’s narratives have been categorized into denial, remedial, and conspiracy discourses, progressively refining its conceptualization [4]. Recognizing its historical precedence, the World Health Organization in September 2020 emphasized the necessity to combat infodemics alongside the pandemic. In the digital age, social media platforms have become central to information dissemination, amplifying the reach and impact of misinformation during crises [5], thus underscoring the urgency of addressing the infodemic.

Scholarly efforts have begun to address the infodemic. The cornerstone of such a mechanism rests on robust government oversight and legal frameworks [6], suggesting a collaborative approach between administrative and legal interventions. Moreover, the varying quality among media practitioners, marked by a deficiency in humanistic values, critical thinking, and global outlook, exacerbates the infodemic. Furthermore, leveraging the strategic value of accurate information, particularly by tapping into public engagement on social media, can refine policy effectiveness. The psychological repercussions of the infodemic on COVID-19 patients are also a significant concern, with the deluge of misinformation contributing to heightened uncertainty in treatment outcomes. Timely psychological interventions have been deemed essential, with studies revealing a correlation between increased social media exposure and psychological distress [7]. Additionally, the application of machine learning and algorithmic analysis in studying infodemic patterns provides empirical insights into topic evolution and public sentiment [8,9]. Advancements in deep learning models have enhanced the detection of misinformation [10], with integrated language and context analysis models proving effective in identifying false narratives [11]. These technological approaches could empower health care institutions to foster informed public discourse during health crises [12].

A review of the literature reveals that current research on the infodemic primarily focuses on the intersection of information governance and health care. While data science and machine learning offer innovative perspectives, comprehensive reviews synthesizing these areas are lacking.

From a bibliometric perspective, the structure of this paper is as follows: The Methods section outlines the methodological framework, detailing the data and methods, with a focus on sample data for empirical analysis. The Results section includes several subsections: temporal trends in domestic and international publications, visual comparative analysis of authorship, institutional contributions, and research directions across countries, as well as a keyword clustering analysis that categorizes domestic and international infodemic research. The Discussion section identifies key challenges for the future of information governance and new media development, exploring each in depth. Finally, the Conclusion concludes with a summary and proposes directions for future research.

## Methods

### Study Overview

This study employed bibliometric methods [13], combined with visualization analysis tools, to systematically map and compare the research landscape of the COVID-19 infodemic at the international and Chinese levels. Bibliometrics is a powerful tool for quantitatively revealing the knowledge structure, evolutionary pathways, and research frontiers within a specific research field [3]. In recent years, this methodology has been widely applied to assess and analyze numerous complex and critical research areas, such as pathways to carbon neutrality [14], the application of artificial intelligence in renewable energy [15], the economic impacts of the COVID-19 pandemic [16], and wastewater treatment for emerging contaminants [17], demonstrating its effectiveness and robustness in mapping interdisciplinary knowledge domains. Therefore, this study utilizes this approach to analyze the equally complex and multidimensional global issue of the infodemic.

Using CiteSpace 5.3, this study systematically searched the Web of Science (WOS) and China National Knowledge Infrastructure (CNKI) core databases, with parameters and outcomes detailed in Table 1. The data visualization timeframe was segmented into 1-year intervals for enhanced clarity.

**Table 1.** WOS^a^ and CNKI^b^ data-related parameter settings.

Related parameter settings	WOS	CNKI
Time limit	2016-2025	
Time slice	1 year	
Retrieval time	Jul 20, 2025	
Node type	The author of the article, the issuing organization, the keywords	
Search method	Subject search	
Retrieve content	“Social media” AND “infodemic”	“infodemic”
Literature resources	Core journal	CSSCI^c^+core journal
Output format	Text format	RefWorks format
Valid data	312	1125

a WOS: Web of Science.

b CNKI: China National Knowledge Infrastructure.

c CSSCI: Chinese Social Sciences Citation Index.

In the WOS database, considering that directly using “misinformation” combined with “social media” yielded limited results, and the term “infodemic” more precisely captures the issue of information overload in the context of COVID-19, we employed a Topic Search with the query: TS=(“infodemic” AND “social media”). In the CNKI database, to comprehensively capture relevant literature, we experimented with multiple search strategies. Both subject searches (Subject=“社交媒体” [social media] AND (Subject=“信息疫情” [infodemic] OR Subject=“错误信息” [misinformation])) and keyword searches (Keyword=“社交媒体” [social media] AND (Keyword=“信息疫情” [infodemic] OR Keyword=“错误信息” [misinformation])) returned limited results. Therefore, we focused on the core theme of “infodemic” and conducted a subject search (Subject=“信息疫情” [infodemic]), as it yielded a larger volume of literature that was highly relevant to our research focus.

The initially retrieved records were screened to ensure relevance and compliance with document type requirements. The inclusion criteria were as follows: document types limited to journal articles (articles or reviews); research content directly related to the infodemic in the context of COVID-19; and language restricted to English (WOS) or Chinese (CNKI). The exclusion criteria were as follows: nonacademic publication types such as news reports, conference abstracts, proceedings, book reviews, notices, and letters; duplicate records; and literature with low relevance to the research topic.

After screening, 312 records from WOS and 1125 records from CNKI were included as the final analytical sample. Complete bibliographic records (including titles, authors, affiliations, abstracts, keywords, and references) were exported in plain text format.

Bibliometrics primarily employs statistical and quantitative techniques to analyze scientific literature. These techniques and their results are summarized in Table 1, referring to the publication timeframe as “time slice.” Publication trends in both domestic and international research are depicted to assess the scope and direction of the field’s evolution. This paper also presents a visual map analysis of author and institution collaborations, offering insights into academic exchange and teamwork patterns. The significance of highly cited works as indicators of scholarly focus is acknowledged [18], allowing us to infer primary academic directions. Current hot topics are identified through cluster analysis of keywords from these highly cited works.

The selection of the WOS Core Collection and the CNKI database was made after careful consideration, aiming to comprehensively cover global research on COVID-19-related infodemics.

WOS, as an internationally recognized premier citation database, indexes high-impact academic literature across multiple disciplines, providing authoritative coverage of international publications.

CNKI, on the other hand, is the most comprehensive and authoritative database for Chinese academic journals, books, dissertations, and conference proceedings, making it indispensable for capturing the substantial body of domestic research on this topic.

### Ethical Considerations

Ethical oversight and approval for this study were provided by the School of Urban Construction at Hebei Normal University of Science and Technology, as the university does not have a dedicated ethics committee for humanities and social sciences research. All procedures were conducted in accordance with the Declaration of Helsinki. Participant data were anonymized and stored securely to protect confidentiality. No compensation was provided.

## Results

### Studies Included

**Figure 1.**
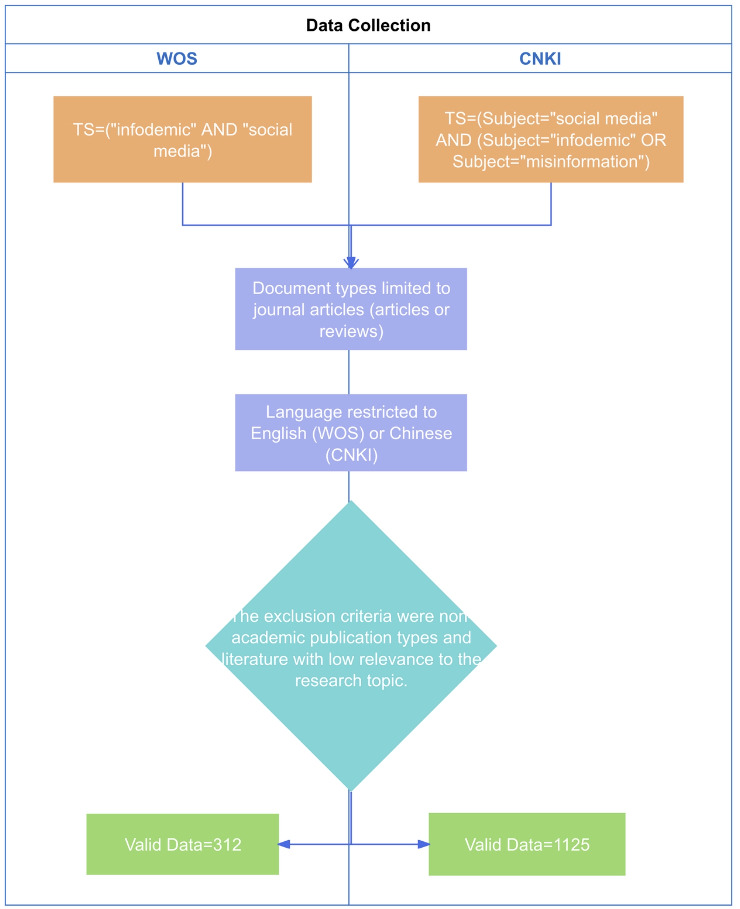
Flowchart of literature screening in WOS and CNKI databases. CNKI: China National Knowledge Infrastructure; WOS: Web of Science.

To illustrate the literature identification and screening process, we created a PRISMA-style flow diagram (Figure 1).

### The Time Trend Analysis

**Figure 2.**
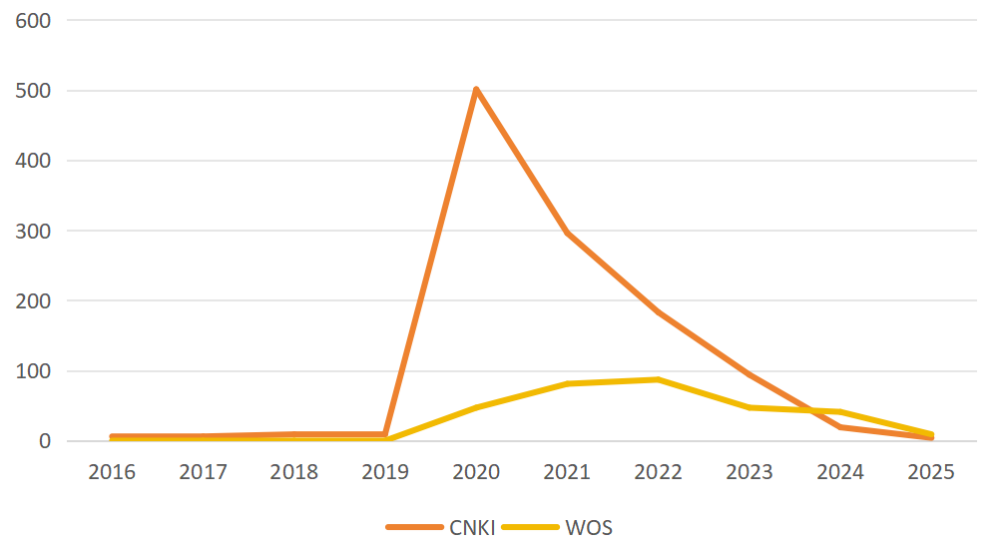
Comparison chart of domestic and international research publications on information epidemics from 2016 to 2025. CNKI: China National Knowledge Infrastructure; WOS: Web of Science.

The volume of publications over time serves as an intuitive indicator of research activity and interest in a field, aiding in forecasting future research trajectories. Analysis of data up to July 20, 2025, reveals 1125 domestic publications and 312 international publications. Figure 2 illustrates a spike in both domestic and international research during 2020 and 2021, followed by a decline, with international publications surpassing domestic output as of July 2025.

The term “infodemic” emerged in response to the misinformation crisis linked to the pandemic, starting in December 2019 [19]. Initially, this concept received limited academic attention, representing a nascent challenge with unclear societal repercussions [20].

The sustained impacts of infodemics have kept scholarly interest alive internationally, as researchers tackle the complex societal issues they create. This ongoing interest is reflected in the steady volume of international research in 2021, characterized by an interdisciplinary approach focused on developing models for infodemic transmission and algorithms for information management [21]. Such cross-disciplinary synergy and technological innovation likely contributed to the growth of international publications.

In 2020, China experienced a turning point as domestic outbreaks underscored the detrimental effects of infodemics. The urgency to combat misinformation spurred a surge in Chinese research, driven by the need for policy development and effective mitigation strategies, resulting in a significant increase in publications that later tapered off with the successful containment of the epidemic [22].

### Analysis of Author Networks

This section analyzes authorship patterns in the field, examining both domestic and international author networks while identifying prolific authors based on their research output. The focus is on authors with multiple publications, exploring themes investigated by collaborative teams through key articles.

Author networks are visually represented by nodes and connections, where each node represents an individual author, and its size indicates the number of publications. Lines between nodes signify collaborative links, with denser lines reflecting more frequent coauthorship. This network analysis provides insights into the level of cooperation and identifies central figures within the research community.

### Global Author Analysis

**Figure 3.**
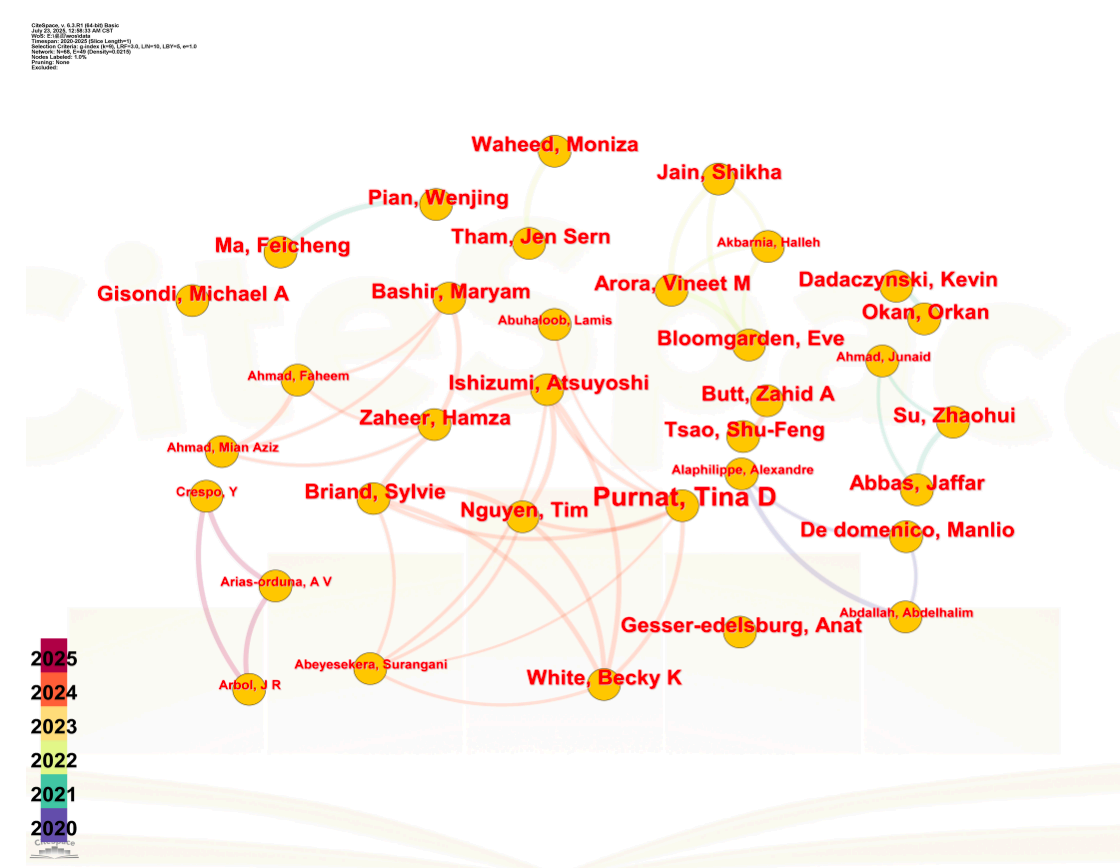
Global author collaboration network for information epidemic research.

This subsection extends the authorship analysis to the international landscape, employing network visualizations to elucidate the collaborative dynamics among global researchers (Figure 3).

The optimized author cooperation network is depicted in Figure 3, generated using CiteSpace software. CiteSpace was developed by Dr. Chaomei Chen, a professor at the College of Computing and Informatics, Drexel University in the United States. The detailed configuration and data information for this part of the CiteSpace analysis are summarized in Table 2, covering key information such as software version and time slice settings. The network consists of 68 nodes (n=68), signifying the involvement of 68 authors, and exhibits a connection density of 0.0215. The sparsity of the connections within the network suggests a low level of collaboration among international authors. Table 3 details articles that have emerged from three distinct collaborative clusters within the network. These articles address a range of topics, including a cross-sectoral information management framework for pandemic response [23], recommendations for social media companies to combat infodemics [24], and novel paradigms for public health communication [25].

**Table 2.** CiteSpace configuration and data information in global author analysis.

Details	Content
CiteSpace v. 6.8.R3 (32-bit)	Software and version
WOS^a^	Data type
2020-2025, slice length=1	Time slicing
g-index (k=25%), RPf^b^=3.0, LN^c^=10, LBY^d^=5, e=1.0	Selection criteria
Largest CC:^e^ 6 (7%)	Largest co-occurrence cluster size
Total nodes: 22 (7.04%)	Total network nodes
Pruning: Pathfinder	Pruning method

a WOS: Web of Science.

b RPf: research publication frequency.

c LN: labeled node.

d LBY: leading board year.

e CC: co-occurrence cluster.

**Table 3.** Foreign authors collaborate on articles.

Writer	Title	Time	Citation frequency
Tangcharoensathien V et al [19]	Framework for managing the COVID-19 infodemic: methods and results of an online, crowdsourced WHO technical consultation	2021	364
Gisondi MA et al [23]	A Stanford conference on social media, ethics, and COVID-19 misinformation (INFODEMIC): qualitative thematic analysis	2022	19
Albrecht SS et al [24]	Lessons learned from the dear pandemic, a social media-based science communication project targeting the COVID-19 infodemic	2022	0

Further analysis of international authors’ publication patterns reveals that several have published two or more papers on the topic. Notably, De Domenico emerged as the most prolific contributor with 4 publications, while 23 other researchers (representing 34% [23/68] of all authors) produced more than two papers each. In terms of collaborative work, the most-cited single paper received 93 citations, demonstrating substantial academic attention and recognition within this research field. This citation impact strongly reflects the high scholarly value attributed to these studies.

### Domestic Author Analysis

**Figure 4.**
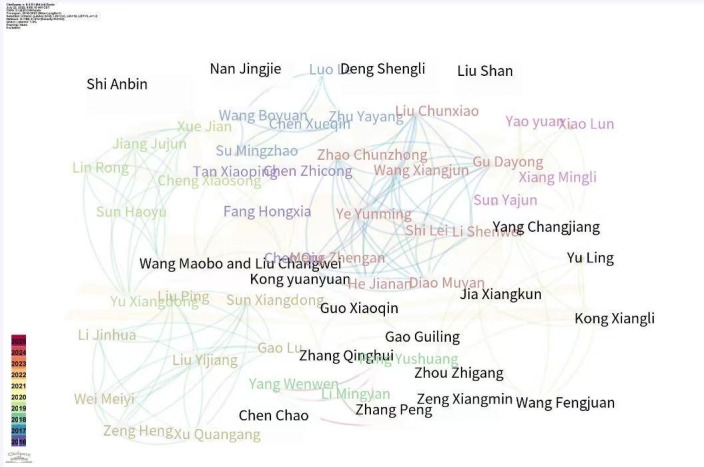
Domestic author collaboration network in infodemic research.

Figure 4 shows the optimized author collaboration network diagram, Table 4 shows the articles written by authors, and the detailed data information from this part of the CiteSpace analysis is summarized in Table 5. There are 168 nodes displayed in the graph (n=168), indicating that 168 people are renting the authors, and the connection density is 0.0153. Small nodes and low density indicate that the author has fewer papers and a low level of cooperation. The map shows only two more complex cooperative networks, led by Wang and Chen [26]. Wang’s group elaborated expert perspectives on addressing infodemics, informing policymaking and platform information dissemination [27]. Li and colleagues probed characteristics and dissemination of scientific information to devise specific countermeasures [28]. Overall, 51 authors have published 2 or more papers, accounting for 30% (51/168) of the total number of authors analyzed. The highest number of citations for a collaborative paper is 3.

**Table 4.** Domestic authors collaborate on articles.

Writer	Title	Time	Citation frequency
Li et al [29]	A cross-sectional survey on information access through hospital’s official account on new media and its association with antenatal care knowledge and health behaviors of pregnant women under COVID-19 epidemics	2021	3
Zong et al [30]	Analysis of Data Collection of Relapsing COVID-19 Epidemic Events in China	2021	1
Li et al [31]	Content characteristics, modes, response strategies and optimization paths of epidemic scientific information dissemination: based on the content analysis of 10 popular microblogs of related scientists	2022	0

**Table 5.** CiteSpace configuration and data information in domestic author analysis.

Details	Content
CiteSpace v. 5.8.R3 (32-bit)	Software & version
2016-2025 (slice length=1)	Timespan
g-index (k=25%), RPf^a^= 3.0, LN^b^=10, LBY^c^=5, e=1.0	Selection criteria
N=168, E=214 (density=0.0153)	Network
Mean silhouette S=0.8898	Silhouette
Harmonic mean (Q, S)=0.7113	Harmonic mean

a RPf: research publication frequency.

b LN: labeled nodes.

c LBY: leading board year.

Identifying core authors is a process that considers both the quantity of output (importance) and the extent of influence (citation counts). The analysis of both domestic and international authors reveals that overall author productivity is modest, with no individuals exhibiting exceptionally high publication rates. Furthermore, the citation rates for the literature are not optimal, suggesting that research into infodemics is still in its formative stages. There is a clear opportunity for researchers to engage in more diverse collaborative efforts, which could significantly enhance the quality and impact of their work.

Core author groups are essential for carrying out in-depth investigations into complex issues, fostering sustained and comprehensive research efforts. Although there are nascent collaborative teams both domestically and internationally, the metrics of importance and influence indicate potential for theoretical expansion and development. This underscores the need for increased interdisciplinary collaboration and more robust research networks to advance the field.

### Institution Analysis

This section evaluates the contribution of various institutions to the field by examining the volume of publications and the extent of interinstitutional collaboration. The analysis of the institutional co-occurrence network, represented through nodes and connecting lines, offers insights into the depth of research on the topic and the collaborative intensity among institutions.

### Foreign Institution Analysis

**Figure 5.**
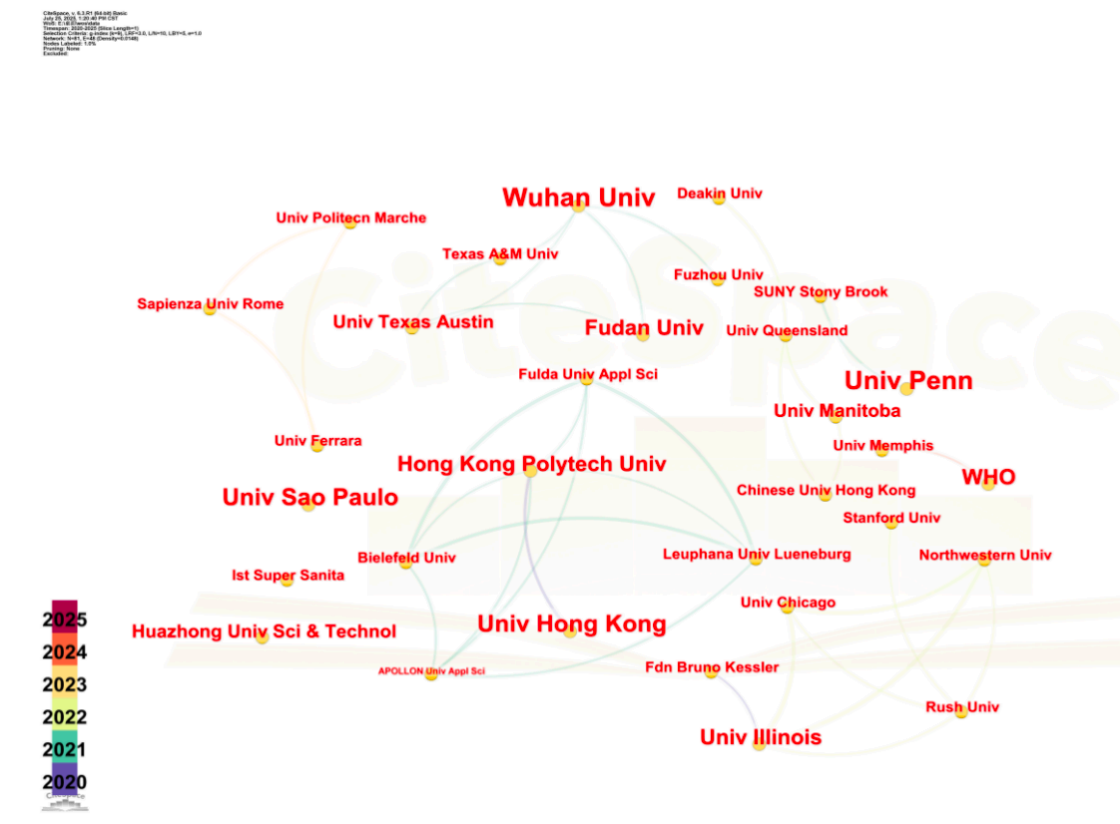
International institutional collaboration network on infodemic research.

The assessment of international institutional engagement is visualized in Figure 5, which illustrates a network comprising 81 entities. The detailed data information of CiteSpace analysis in this section is presented in Table 6, including key parameters such as time span and selection criteria, providing support for the reliability of network analysis. The network exhibits a slightly higher level of connectivity (density=0.0148) compared to domestic institutions, indicating a modest degree of international collaboration. Predominantly universities, these foreign institutions demonstrate greater collaborative activity than their domestic counterparts, though there is still considerable room for deepening these cooperative relationships.

**Table 6.** CiteSpace configuration and data information in foreign institution analysis.

Details	Content
CiteSpace v. 5.8.R3 (32-bit)	Software & version
2020-2025 (slice length=1)	Timespan
g-index (k=25%), RPf^a^=3.0, LN^b^=10, LBY^c^=5, e=1.0	Selection criteria
N=73, E=54 (density=0.0207)	Network
Largest CC^d^: 7 (9%)	Largest co-occurrence cluster size
Total nodes: 16 (21.9%)	Total network nodes

a RPf: research publication frequency.

b LN: labeled nodes.

c LBY: leading board year.

d CC: co-occurrence cluster.

Wuhan University emerges as the most prolific contributor with six published articles, having established collaborative links with Fudan University and Fuzhou University, each on two occasions. Despite these partnerships, the depth of international cooperation, as reflected by the overall network density, remains limited.

Further analysis of the data from institutions publishing international papers reveals that among those publishing four or more international papers, Wuhan University stands out with six papers. Its research focuses on the characteristics, transmission mechanisms, and network information flow regulation strategies of information epidemics. Notably, one of the university’s papers has been cited seven times. Through media type analysis, it quantifies the spatial correlation between information epidemics and actual epidemics, finding that mobile media may increase the transmission risk coefficient. Other major institutions include Oxford University, Hong Kong Polytechnic University, Bruno Kessler Foundation, and the University of Pennsylvania, all of which have published four significant research outcomes.

### Domestic Institution Analysis

The visualization of domestic institutions indicates 209 contributing entities (n=209), yet the network’s density is 0.0028, signifying no discernible connections between them. The modest number of publications from each institution results in smaller nodes and an absence of a visible network, suggesting a nascent stage of institutional collaboration in this research area.

Among domestic institutions, 76 have published two or more papers, primarily universities and research institutions. As the core force in academic research, these institutions continue to explore this emerging social issue; from a geographical perspective, they are widely distributed across China, reflecting the widespread attention of the domestic academic community to this field.

The School of Information Management at Nanjing University has published 10 papers, making it the university with the highest number of published papers in China. The Communication University of China stands out with seven publications, underscoring its significant role in examining information dissemination and governance. One notable publication from this institution delves into media users’ characteristics, cognitive assessments, and interactions, providing theoretical insights for governance.

The fact that 133 institutions have contributed only a single paper each highlights a lack of sustained research efforts across most entities. The absence of connectivity within the network (density=0) indicates that research efforts are isolated, with institutions working independently rather than collaboratively. This isolation suggests potential for greater cooperation, which could foster more comprehensive studies and a deeper understanding of the issues at hand.

In conclusion, while the foundations for international institutional cooperation are in place, the impact and breadth of this collaborative effort require enhancement. It is important to recognize the multidisciplinary nature of infodemic research, which intersects with journalism, communication, computer science, public management, and other fields. Sustained and strategic collaboration among institutions is pivotal for in-depth exploration of the topic and for elevating the quality of research outputs. Therefore, there is a clear need to fortify the collaborative ties between research institutions and to facilitate interdisciplinary communication to advance the study of information epidemics effectively.

### Highly Cited Literature Analysis

The frequency of citations serves as a barometer for scholarly impact, with highly cited papers often indicative of their influence and prominence within the academic community. An in-depth examination of citations and keywords from these seminal works provides insight into the prevailing research themes and potential future directions.

### Foreign Highly Cited Literature Analysis

Table 7 contrasts the domestic citation landscape with the international scene, which demonstrates a broader academic reach. Keywords such as “COVID-19,” “social media,” “mental health,” “depression,” “public attention,” and “communication content” dominate these papers, reflecting concerns over the spread of misinformation on social media during the pandemic. The synthesis of keywords from these influential international articles reveals focal areas of global concern: the mental health impact of the infodemic [32], the development of infodemic response strategies and various facets of information governance, including thematic communication and evolution [33,34], as well as monitoring and evaluative frameworks [35,36].

**Table 7.** Top 5% citation frequency in WOS.^a^

Number	Title	Citation frequency	Keywords
1	Mental health problems and social media exposure during COVID-19 outbreak [6]	595	COVID-19; mental health; social media
2	The COVID-19 social media infodemic [32]	274	epidemiology; computer science; information theory; and computation
3	COVID-19–related infodemic and its impact on public health: a global social media analysis [34]	192	COVID-19; infodemic; impact on public health
4	Mental health, risk factors, and social media use during the COVID-19 epidemic and Cordon Sanitaire among the community and health professionals in Wuhan, China: cross-sectional survey [37]	181	COVID-19; social media; population mental health; depression
5	Coronavirus (COVID-19) "infodemic" and emerging issues through a data lens: the case of China [38]	178	COVID-19; infodemic; humanitarian emergency
6	The impact of social media on panic during the COVID-19 pandemic in Iraqi Kurdistan: online questionnaire study [25]	175	social media; COVID-19; infodemic; mental health
7	COVID-19 infodemic; more retweets for science-based information on coronavirus than for false information [35]	151	communicative content analysis; infodemic; social media analytics
8	Framework for managing the COVID-19 infodemic: methods and results of an online, crowdsourced WHO technical consultation [19]	94	COVID-19; infodemic; knowledge translation; message amplification; misinformation; risk communication
9	Chinese public attention to the COVID-19 epidemic on social media: observational descriptive study [39]	94	public attention; social media; public health emergency
10	Assessing the risks of “infodemics” in response to COVID-19 epidemics [33]	92	COVID-19; infodemic; risk

a WOS: Web of Science.

In summary, while the domestic articles are gaining traction, international literature, with its broader citation reach, highlights a global engagement with the intricate challenges posed by the infodemic. This underscores the necessity for a concerted, multidisciplinary approach to understand and mitigate the effects of misinformation, especially in the context of global health crises.

### Foreign Highly Cited Literature Analysis

As of the data collection cutoff, domestic literature exhibits a relatively modest citation footprint. However, it is important to acknowledge the potential for a rise in citation frequency for more recent publications. Table 8 lists the three most cited domestic articles, with citation counts of 49, 45, and 35, respectively. A keyword analysis of these leading papers reveals primary research themes among the highly cited domestic literature: infodemic response strategies, information governance mechanisms, and patterns of information dissemination.

**Table 8.** Citation frequency in CNKI.^a^

Number	Title	Citation frequency	Keywords
1	Analysis and Countermeasures of Problems in China's Disease Prevention and Control Information System [40]	49	Epidemic control; public health emergencies; intelligence system
2	What kind of public information dissemination do we need to build? Reflections on new media communication during the COVID-19 pandemic [41]	45	Pandemic; public information dissemination; social media; decentralization; epidemic information
3	Association between digital media use and anxiety symptoms of college students during the novel coronavirus pneumonia epidemic [42]	35	Epidemic prevention and control; media use; information needs; media trust; information governance; public emergency
4	Integrated management of the COVID-19: practical test and optimization path [43]	34	Public health emergencies; COVID-19; online public opinion; information disclosure; multiple subjects
5	Legal protection of personal information in major epidemic prevention and control [44]	34	Personal information; epidemic prevention and control; data against the epidemic; information protection; use the information
6	Population movement, information transmission efficiency, and epidemic control: evidence from COVID-19 [45]	29	COVID-19; population mobility; information transmission efficiency; brand theory
7	Personal information protection in public health events -- from the perspective of COVID-19 prevention and control [46]	29	COVID-19; information privacy; public interest; proportionality
8	Research on Risk Perception and Risk Transmission Models in Public Health Events: A Discussion on the Moderating Effect of Epidemic Severity [47]	28	Risk information; risk perception; communication behavior; epidemic severity
9	Use of personal information and legal rules in public emergencies: COVID-19 response as a starting point [48]	28	Emergency response; use of personal information; the rule of law

a CNKI: China National Knowledge Infrastructure.

### Keywords Clustering Analysis

Keyword clustering is a methodological approach that groups semantically related keywords to identify dominant themes within a body of literature. This analytical technique can provide insights into the central topics of research by examining the centrality, frequency, and co-occurrence of keywords. In this section, the software CiteSpace is utilized to conduct a clustering analysis of the literature, with the aim of highlighting distinctions between domestic and international research foci.

### Foreign Keywords Clustering Analysis

**Figure 6.**
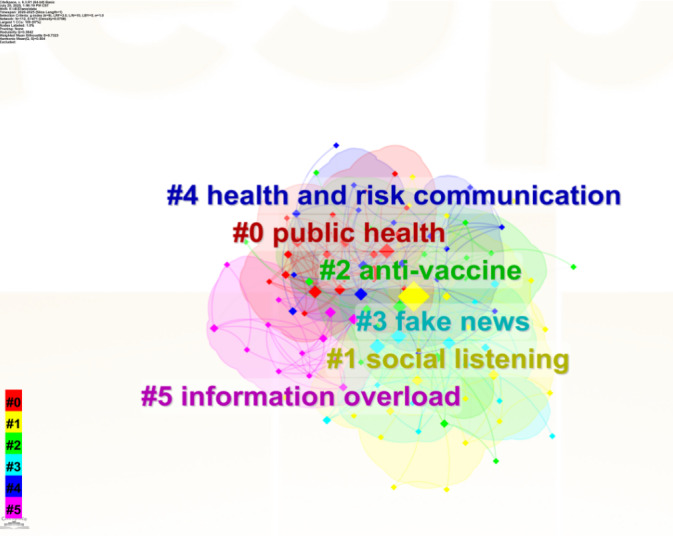
Thematic evolution of global infodemic research.

The data information regarding the keyword clustering analysis of foreign literature in this section is detailed in Table 9. To facilitate a clearer representation, the “Convex Hull: Show/Hide” feature in CiteSpace can be employed to display the clustering outcomes as distinct colored shapes. The modularity value Q and the mean silhouette value S are recognized as key indices for evaluating the quality of clustering. A Q value greater than 0.3 is indicative of a well-defined structure characterized by high internal consistency and relatedness, while an S value approaching 1 suggests an optimal clustering scale. Internationally, the Q value is 0.3842, S value is 0.7323, and the mean of Q and S is 0.5583, as shown in Figure 6. The clustering results for both domestic and international literature denote clear demarcations and appropriate scales, affirming the reliability of the cluster analysis.

**Table 9.** CiteSpace configuration and data information in foreign keywords clustering analysis.

Details	Content
CiteSpace v. 5.8.R3 (32-bit)	Software & version
2020‐2025 (slice length=1)	Timespan
g-index (k=25%), RPf^a^=3.0, LN^b^=10, LBY^c^=5, e=1.0	Selection criteria
n=112, E=471 (density=0.0758)	Network
Largest CC^d^: 109 (97%)	Largest cluster (CC)
Modularity Q=0.3842	Pruning: Pathfinder
Mean silhouette S=0.7323	Mean silhouette
Harmonic mean (Q, S)=0.504	Harmonic mean (Q, S)

a RPf: research publication frequency.

b LN: labeled nodes.

c LBY: leading board year.

d CC: co-occurrence cluster.

### Domestic Keywords Clustering Analysis

**Figure 7.**
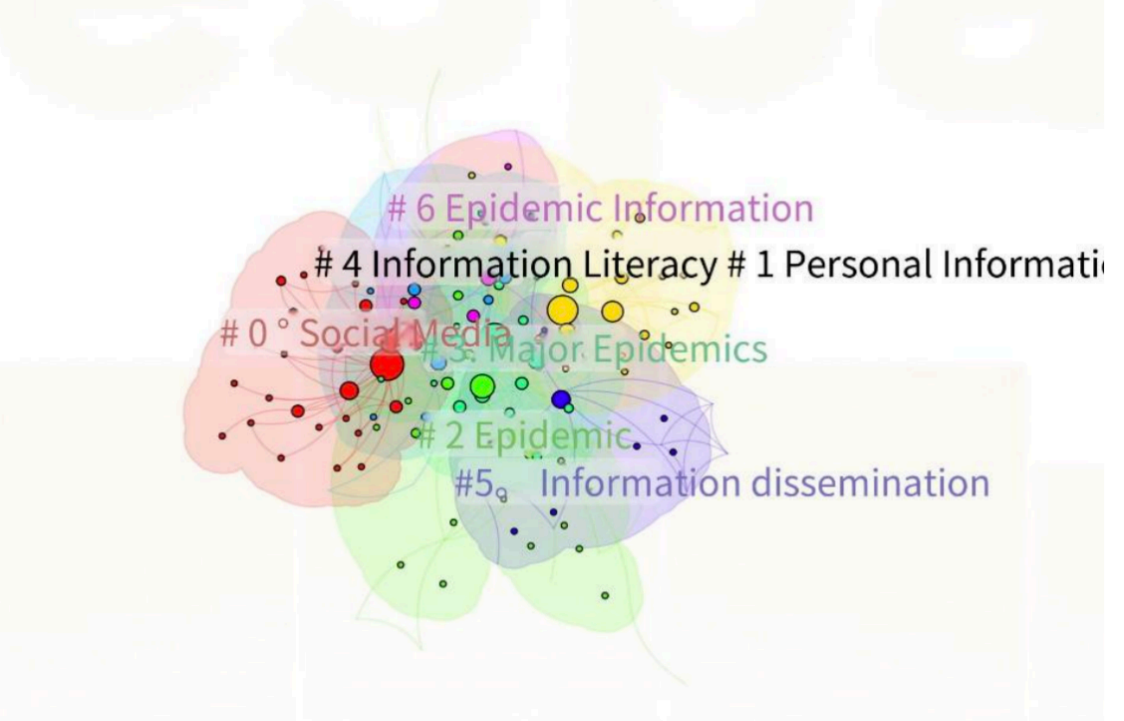
Thematic hotspots distribution in China’s infodemic research.

The domestic literature exhibits a Q value of 0.4715 and an S value of 0.7964 (as shown in Table 10), with an average of Q and S at 0.6339 as depicted in Figure 7.

**Table 10.** CiteSpace configuration and data information in domestic keywords clustering analysis.

Details	Content
CiteSpace v. 5.8.R3 (32-bit)	Software & version
2016‐2025 (slice length=1)	Timespan
g-index (k=25%), RPf^a^=3.0, LN^b^=10, LBY^c^=5, e=1.0	Selection criteria
n=145, E=398 (density=0.0381)	Network
Largest CC^d^: 122 (84%)	Largest cluster (CC)
Nodes labeled: 1.0%	Nodes labeled
Modularity Q=0.4715	Modularity
S=0.7964	Weighted mean silhouette
0.5923	Harmonic mean (Q, S)

a RPf: research publication frequency.

b LN: labeled nodes.

c LBY: leading board year.

d CC: co-occurrence cluster.

Further, to delve into the specifics of the keyword clusters, this section examines the predominant keywords within each cluster. Clusters are assigned serial numbers, with smaller numbers indicating a larger scale and a higher number of keywords. CiteSpace analysis of the CNKI and WOS databases yielded 9 and 8 keyword clusters, respectively. Based on clustering intensity, the top six clusters were identified for further scrutiny. The “Cluster Explorer” function in CiteSpace calculates the intensity of topics, and the high-frequency keywords within each cluster are summarized to capture the essence of the research themes, as indicated in Table 11.

**Table 11.** Classification and intensity of foreign and domestic literature themes.

No	WOS^a^ topics’ high frequency words	Intensity of themes	WOS topics	CNKI^b^ topics	Intensity of themes	CNKI topics’ high frequency words
0	Social media; Twitter; behavior; attitude; online	0.757	News	Information literacy	0.924	Epidemic prevention and control; emergency management; information technology; integrated media
1	Information; communication; Facebook; impact; knowledge	0.746	Fake news detection	Information spreading	0.978	Social media; opinion leaders; risk communication; response strategies
2	Health; adult; care; virus; support	0.812	Machine learning	Message protection	0.835	COVID-19; mainstream media; information disclosure; personal information
3	Media; misinformation; risk; quality	0.86	Social network	Infodemic	0.858	Information overload; information explosion; political epidemic; public opinion guide
4	Fake news; diffusion; science; anger	0.711	Mortality salience	Major epidemic disease	0.785	Interest measurement; proportionality
5	Trust, depression, scale, need, belief, anxiety	0.765	Segmentation	Media trust	0.954	Information governance; media needs; media use; source of information

a WOS: Web of Science.

b CNKI: China National Knowledge Infrastructure.

The keywords encapsulate the essence of the articles’ content and mirror the research directions. Through the keywords clustering analysis, this study categorizes the research domains of the infodemic both domestically and internationally into six themes: (1) Domestic clusters #1 and #3 and international clusters #0 and #3 are focused on the dissemination of information. (2) Domestic clusters #0 and #4 are primarily concerned with utilizing information for COVID-19 control. (3) Domestic clusters #4 and #5 underscore the importance of information governance. (4) Domestic cluster #2 is dedicated to the protection of information. (5) International clusters #1 and #2 are centered on machine learning and algorithm development. (6) International clusters #4 and #5 highlight the need for clinical attention to mental health issues stemming from the epidemic, advocating for prompt and effective psychological interventions. The subsequent sections will provide a detailed analysis of these identified research directions.

### Information Spreading

The rise of the internet and digital technology has transformed information dissemination, necessitating a reevaluation of distribution methods in contemporary society. The interaction between traditional and social media has become central to this transformation, with researchers suggesting that they can complement each other. Integrating new media technologies with professional journalism principles creates a synergistic opportunity for enhancing information spread.

However, the complexity and unpredictability of online communication, along with public engagement with new media, contribute significantly to the proliferation of the “information plague.” In this context, it is vital that the social responsibilities of social media platforms are not overshadowed by their technological capabilities.

To effectively regulate information spread, it is crucial to monitor and address the dissemination of false information while understanding its evolving patterns. Research indicates that trends in misinformation often align with the trajectory of the pandemic, with themes developing in tandem. The role of influential figures and institutions in propagating misinformation is significant; for instance, former President Donald Trump played a key role in amplifying COVID-19 vaccine conspiracy theories on Twitter [49,50]. This underscores the need for new methods to regulate and standardize information dissemination.

Furthermore, inadequate evaluation and oversight of information releases have contributed to misinformation spread. Initiatives like WeChat’s rumor evaluation system have effectively curbed certain false narratives [21]. These examples demonstrate the potential for systematic approaches to mitigate misinformation risks in the digital age.

### Using Information to Control COVID-19

During the COVID-19 pandemic, information has emerged as a critical tool for maintaining social stability and managing public health emergencies. A nation’s ability to respond effectively to such crises depends on its governance and strategic information dissemination. Empirical evidence indicates a positive correlation between public trust in information and adherence to preventive measures, highlighting the importance of leveraging information to control the virus’s spread.

In response, some scholars have proposed an innovative model for information communication designed to enhance interaction among stakeholders. This model could improve public policy quality and reduce resistance during implementation [23,51], while also boosting public confidence in official agencies and media [52].

Research shows that online information resources are crucial for governments to rapidly assess public health needs and refine health interventions [53]. Practical experiences demonstrate that managing the COVID-19 crisis requires coordinated efforts among diverse stakeholders. Information resources play a pivotal role in bridging departmental gaps, fostering consensus, and establishing comprehensive response mechanisms, thereby expediting epidemic prevention and control. The mainstream media, particularly official outlets, is critical for promptly disseminating accurate information, shaping public opinion, and fostering a unified societal response.

These findings illustrate that information has become an invaluable resource for governmental governance during the pandemic.

While countries have adopted various strategies to combat COVID-19, the universal objective remains effective control guided by humanitarian principles. The government’s role is central to navigating public crises, and the misuse of information for political gain undermines global efforts against the epidemic. The politicization of the pandemic, as evidenced by contentious social media discussions on issues like racism, can detract from a unified response. Therefore, adherence to scientific principles, judicious policymaking, and harmonizing government and societal efforts are essential to prevent the politicization of the pandemic response.

### Information Governance

The concept of information governance has gained heightened significance following the COVID-19 pandemic, particularly regarding the regulation of information dissemination and the role of social media. Misinformation has demonstrated a lasting and detrimental impact on social trust, often surpassing the effects of the virus itself, which underscores the urgent need for stringent governance by authorities. A key governance strategy is the responsibility of official media to swiftly provide accurate news, thus countering the spread of falsehoods.

At the heart of information governance is oversight. Researchers assert that timely dissemination of factual information can significantly mitigate information asymmetry, necessitating a greater sense of responsibility among information publishers and a reinforced commitment to ethical and legal standards. Misinformation not only incites public panic and erodes societal trust but also challenges the rapidly evolving social media landscape. Addressing these challenges requires targeted legislation to regulate information publication on social media platforms.

The improvement of the institutional framework serves as the cornerstone for the systematic and standardized operation of information governance. Given the complexity and uncertainty in the current information environment, there is an urgent need to establish a regulatory policy and standard system that is structured, clear in terms of responsibilities, and dynamically adjustable. First, it is imperative to strengthen top-level design and formulate forward-looking and inclusive national or regional information governance strategies. Second, targeted and highly operable special regulations should be introduced to provide a solid legal basis for governance practices. Simultaneously, promoting cross-departmental and cross-disciplinary policy coordination is crucial. It is essential to break down information barriers and establish unified standards for information quality assessment, content review norms, and crisis response plans to ensure consistency and effectiveness of governance actions. Lastly, the formulation and promotion of standards are indispensable. It is advisable to encourage the development and adoption of internationally and domestically recognized technical and management standards for information security, privacy protection, data interoperability, etc, providing a unified scale and a basis for mutual trust for the development and application of information governance tools.

The iterative upgrading of technological tools is a key driving force for enhancing the efficiency of information governance. In the face of the explosive growth and complex evolution of information epidemics, it is imperative to fully tap into and integrate the potential of cutting-edge technologies. At the information recognition level, we should deepen the application of artificial intelligence and natural language processing technologies, develop multimodal (text, image, audio, and video) content understanding capabilities, and enhance the efficiency of automated and precise identification of false, misleading, and harmful content in massive amounts of information, with particular attention paid to the analysis of implicit intentions and emotional tendencies at the semantic deep level.

The experience of managing the COVID-19 crisis has underscored the necessity of robust information governance for governments. Recognizing it as a systematic endeavor, establishing a professional intelligence team is essential. Relevant departments must align information science development with practical needs, implement a national security strategy informed by big data analytics, and encourage interdisciplinary collaboration supported by technological advancements. In this process, it is crucial to improve the institutional framework and technical support for information governance. By clarifying the rights and responsibilities of each subject and optimizing regulatory technical tools, we can ensure the authenticity and security of information dissemination, providing a standardized and orderly environment for information exchange among different cultures, countries, and civilizations around the world.

### Information Protection

The use of big data technologies for epidemic prevention—such as disease monitoring and population flow management—has become widespread. However, the associated risks of personal information exposure and data breaches necessitate a robust information protection strategy.

To mitigate these vulnerabilities, a new systemic approach to prevent information leakage is essential. The rise of digital technologies calls for a reevaluation of traditional ethics and regulations, particularly in balancing individual rights with societal needs and harmonizing social governance with privacy protection. Research indicates that an institutional framework allowing extensive data collection alongside stringent usage controls (a “wide entry and strict exit” model) can help reconcile public and private interests.

Furthermore, prioritizing the development and implementation of technologies to prevent unauthorized data access is critical. Creating specialized storage platforms for sensitive information will aid in developing standards and protocols for data usage. Additionally, enhancing public awareness of personal information security and clarifying the responsibilities of data collectors are urgent.

In summary, comprehensive protection of personal information relies on sustained engagement from multiple stakeholders. This involves establishing systematic data collection and storage, rigorously assessing data usage, and implementing traceability measures to ensure accountability.

### Machine Learning

The divergence in machine learning application between domestic and international research is notable, particularly in the realm of information governance. Global studies extend beyond the technical manipulation of social media, delving into the intricate layers of information management.

Machine learning emerges as a critical tool where traditional information technologies falter, especially in curtailing the proliferation of misinformation [54]. The intersection of big data and machine learning is a breeding ground for innovation, with algorithms spearheading theoretical advancements. Empirical evidence underscores the efficacy of techniques like the Synthetic Minority Over-sampling Technique and Classifier Vote Ensemble in identifying and curbing the spread of false narratives [55].

In the context of Twitter, Latent Dirichlet Allocation models have been instrumental in sifting through data to extract prevalent topics and sentiments, thereby mitigating the adverse effects of negative discourse on COVID-19 management. Elhadad and Gebali [56] have harnessed machine learning to create an integrated information management system on Twitter, bolstering data protection and reducing misinformation. Similarly, Lu [57] developed a health belief model to quantify health information, promoting the dissemination of constructive content to users. Domestic research has conducted algorithm adaptation based on local data characteristics in the application of such technologies, such as optimizing text classification models for Chinese semantic complexity, analyzing short video content for rumor identification during the pandemic, and accurately disseminating health knowledge on mainstream social platforms based on user personas.

Platforms like WhatsApp have been leveraged by entities such as the WHO and governmental bodies to disseminate verified information rapidly across demographics, capturing the pulse of public sentiment [58]. Machine learning’s capacity to assimilate societal feedback epitomizes the concept of ‘social listening,’ refining policy relevance and efficacy based on public input, thus facilitating smoother policy implementation [59]. The Chinese government utilizes a multimodal collaborative governance system, including the China Internet Joint Rumor Refutation Platform, intelligent public opinion monitoring system, etc, combined with a three-tier public opinion response mechanism, to improve the efficiency of information content governance.

Overall, global research is highly convergent in terms of underlying machine learning technologies, but application paradigms vary due to social governance structures and data ecosystems. International research focuses on algorithmic innovation for open platforms, while domestic practices emphasize multisystem collaborative governance. These investigative efforts converge on the objective of harnessing machine learning to streamline information dissemination, with a strong underpinning in computer science, all while aiming to alleviate the detrimental effects of the pandemic and accelerate the practical testing and iterative optimization of these technologies during the pandemic.

### Psychological Intervention

The domain of psychological intervention distinguishes itself in the dichotomy of domestic and international research orientations. Prompt psychological interventions have emerged as innovative strategies for supporting COVID-19 patients’ mental health. The “infodemic” associated with the pandemic often triggers mental health challenges like anxiety and depression [60]. Evidence indicates that youths with extensive media consumption are particularly susceptible to psychological distress, including anxiety. Moreover, the barrage of negative information prevalent on social media platforms can disrupt public sleep patterns, adversely affecting patient recovery [61].

Research reveals that under similar stressors, psychological resilience tends to be more robust in males. Timely psychological interventions, akin to mental “inoculations,” have proven effective in bolstering individuals’ resilience to negative information. It has also been observed that psychological responses vary across different stages of an information epidemic; hence, interventions should be tailored to these specific stages to optimize mental health outcomes [62]. This insight advocates for a clinical approach that integrates psychological support with traditional medical treatments, emphasizing the necessity for regulatory bodies to monitor and manage media narratives [63]. These findings underscore the practical importance of research in information dissemination and governance.

Furthermore, machine learning can play a pivotal role in information-based psychological interventions. Enhancing the credibility and authenticity of online information through machine learning algorithms has the potential to decrease morbidity [64]. A healthy social network platform developed connects patients with medical professionals, leveraging intelligent algorithms to amplify beneficial health information and counteract negative influences. Addressing societal psychological challenges requires a comprehensive approach that merges epidemic management with the establishment of dedicated psychological organizations to undertake community mental health initiatives [65]. Notably, the research on psychological interventions increasingly intersects with the themes of machine learning, information dissemination, and governance.

## Discussion

### Overview

In light of the pervasive information challenges posed by the global novel coronavirus pneumonia pandemic, this paper posits five critical challenges for the future of information governance and the development of new media. These challenges underscore the need for robust, adaptive frameworks capable of navigating the complexities of information dissemination and management in an era defined by rapid technological advancement and global interconnectedness. This study systematically captures the multiagent interaction characteristics of the information epidemic from multiple perspectives and simultaneously responds to the WHO’s call for “interdisciplinary collaboration gaps,” combining domestic practical experience to deepen the understanding of governance complexity. The insights garnered from this study not only contribute to academic discourse but also serve as a guiding beacon for policymakers, media professionals, and researchers striving to fortify societal resilience against the pernicious effects of misinformation in the digital age.

### Enhancing Collaborative Networks Among Scholars and Institutions

The study’s findings highlight a significant gap in knowledge exchange among domestic and international scholars and institutions, hindering effective responses to emerging challenges. The absence of a prominent cohort of authors and institutions in this discourse underscores the need for a more unified and collaborative approach to address the information epidemic. Publication trends indicate a decline in academic output following an initial surge, suggesting that while research on the information epidemic spans six main domains, it remains in an embryonic stage, with considerable potential for further exploration.

To advance the field, scholars must move beyond isolated research practices and engage in active collaboration with peers. Establishing research networks and sustainable communication channels will facilitate a more comprehensive exploration of complex issues, foster the development of authoritative research leaders and teams, and generate practical solutions for societal advancement and governance. Academic institutions need to step out of the theoretical ivory tower and pay attention to real-world issues in practice; enterprises should open up their technological resources to participate in public issue research; and the government needs to build a neutral collaboration platform. This collaboration is not simply a matter of resource accumulation but rather the formation of a governance synergy through complementary knowledge. In particular, in the context of rapid changes in the information ecosystem, a flexible network structure is more adaptable to demand than a fixed mechanism.

Given the interdisciplinary nature of the infodemic, which intersects journalism, communication, computer science, and public governance, there is an urgent need to create cross-disciplinary communication frameworks that transcend temporal and spatial barriers. Research entities should organize regular academic forums to encourage interaction among experts from various fields. Additionally, online platforms can play a crucial role in achieving consensus, shaping actionable and educational methodologies, and enhancing the quality of scholarly contributions.

### Fostering Expert Teams for Advanced Media Intelligence

The analysis emphasizes the critical importance of information governance and strategic information utilization in mitigating COVID-19, identifying these as key research areas. Post-pandemic reflections underscore the need to assemble skilled professional teams to effectively manage the information epidemic and enhance information management systems. Despite progress in forming domestic intelligence teams, a significant gap remains between big data analysis and its practical application. The efficiency bottleneck of information governance largely stems from the capability gap among professionals: traditional media practitioners lack the ability to apply technological tools, while technical experts often overlook the social impact of information dissemination. This gap leads to a disconnect between technological innovation and actual governance needs. The rapid evolution of digital technologies necessitates a reevaluation of talent development programs, innovative cross-disciplinary training models, and alignment of educational outcomes with societal needs.

Higher education institutions should restructure their curriculum systems by breaking down disciplinary barriers. For instance, they are well-positioned to cultivate multidisciplinary talent by adopting “journalism+ X” approaches that enhance journalists’ skills in data acquisition, processing, analytics, and digital media operations. Educational policymakers should implement a dual-track system that fosters both specialized and technologically proficient professionals. Integrating computer science with social sciences, such as journalism, can enrich curricula and enhance students’ practical experience.

For industry professionals, comprehensive vocational training through expert-led workshops, digital courses, and certification programs is essential to equip the workforce with the skills necessary for managing intelligent media technologies and operations.

Simultaneously, news media must uphold their fundamental societal role. Media professionals should remain vigilant to the challenges of the digital landscape and strive to deepen their humanitarian insights. News content creation must reflect an understanding of diverse cultures and foster global citizenship. With the diversification of communication channels and social platforms, there is an urgent need for a future workforce skilled in advanced media technologies and operational competencies, capable of engaging international audiences while maintaining the humanistic ethos central to journalism.

### Refining the Public Emergency Information Management System and Bolstering Personal Data Protection

The core contradiction faced by information management in public emergencies is the balance between efficient information flow and personal privacy protection. Advancing a public emergency information management system requires a dual focus on efficient information gathering and stringent protection of that information. The system must enhance response capabilities to information risks, supported by technological tools, standardized security measures, and privacy protections. Employing machine learning and algorithmic techniques is crucial for data assimilation, processing, and analysis, enabling informed engagement in societal governance. Tools such as health codes and information flow tracking have proven instrumental in managing the COVID-19 crisis.

In the digital age, intelligence analysis has shifted toward a big data-centric approach, emphasizing the need to understand the interactions between online and offline elements within information systems. This involves harmonizing online theoretical advancements with offline practical applications. Additionally, machine learning and algorithms can provide insights into the psychological responses of different social demographics during emergencies, enhancing intelligent management capabilities.

Establishing standardized information protocols is foundational to an effective management system. For example, China’s initiative to develop a health information standard framework could serve as a model for creating standardized digital technology protocols, data security measures, and benchmarks across various sectors.

To improve the digital emergency information system, it is essential to address the overcollection of personal data and strengthen personal information protection [66]. Technological advancements should elevate information security protocols, including routine data anonymization, to prevent unauthorized access and the development of encryption and real-time monitoring systems.

At the legislative level, robust laws are critical for information security. Clearly defining the boundaries of information collection, analysis, and use while adhering to ethical and legal principles is vital. Regulatory authorities should implement safety protocols to ensure secure information practices.

From the public’s perspective, raising awareness about information security is paramount. Individuals should understand their legal rights and engage with digital services with vigilance and informed caution.

### Enhancing Public Information Literacy and Psychological Resilience

The spread of the information epidemic is partly attributed to the public’s limited skills in observing, analyzing, and evaluating information. Active public participation in information governance is crucial to addressing this challenge. In an information-overloaded environment, the public is prone to experiencing “information anxiety” and may even reduce their trust in authoritative information. Evidence suggests that areas with lower reliance on social networks and reduced trust in online information have experienced lower mortality rates, indicating that the psychological effects of the information epidemic may be more difficult to manage than the pandemic itself.

To enhance the public’s information discernment capabilities, a multifaceted and ongoing approach is essential. Educational initiatives in science can provide timely and factual information about current issues, establishing a robust mechanism for public awareness. This can be achieved through traditional seminars and online platforms, utilizing short videos, social media, and interactive app-based Q&A sessions to create an engaging information dissemination network.

Equally important is the development of critical thinking skills, enhanced information literacy, and a judicious approach to consuming information. These steps are vital for mitigating the negative impacts of the information epidemic on individuals and society.

### Enhancing Media Accountability and Directing Public Discourse Positively

An effective information dissemination environment is essential for managing the information epidemic, a concern shared by scholars globally. Strengthening media accountability requires advocating for the rule of law. In response to the evolving digital media landscape, authorities have implemented laws and regulations to support healthy sector growth. Administrative oversight must involve nuanced management, imposing sanctions that align with the impact of misinformation and its divergence from the truth. Timely release of authoritative information is vital for shaping public opinion and countering misinformation with verified facts.

During emergencies, establishing a direct reporting system is crucial for enhancing information dissemination speed. Legislative amendments have improved the transparency of professional institutions, streamlined the information approval process, and facilitated timely information release during crises.

The timely release of authoritative information is crucial for guiding public opinion. State media should create a robust platform for information dissemination, leveraging social media to integrate official and community channels. Furthermore, cultivating a culture of legal and ethical awareness within the media is indispensable. Media professionals must prioritize information refinement and quality throughout the news production cycle, enhancing fact-checking and peer review processes to ensure authenticity and neutrality [67].

Media personnel should strive for accurate and prompt translation services, maintain objectivity, and minimize biases from political, cultural, or commercial influences to prevent the information epidemic from escalating into a political crisis. Additionally, industry professionals must elevate ethical standards, adopt a people-centric approach, and enhance their expertise through in-depth interviews with scientists and similar initiatives to improve public access to scientific information.

### Research Limitations

There are several limitations in this study that need to be objectively explained: the research perspective is mainly based on the analysis of domestic information governance practices, and there is insufficient comparison of information governance models under different countries’ social systems and cultural traditions, which may limit the cross-contextual applicability of the theoretical framework. The analysis focuses on the construction of macro governance mechanisms, and the exploration of micro-psychological motivations for individual information behavior is not deep enough, making it difficult to fully explain the complex decision-making logic of the public in information selection. The research method focuses on qualitative analysis and framework construction. Although some practical cases are combined, there is a lack of dynamic verification of governance effectiveness through long-term tracking data, and the timeliness of the conclusions needs further observation. These limitations provide directions for improvement in future research, which can be improved through cross-border comparative studies, microbehavioral experiments, and long-term follow-up surveys.

### Potential Research Directions

Our research has found that there are differences in research between the East and the West in related fields, which provides many perspectives for future research.

First, a cross-cultural comparative framework can be established to study the different effects of information governance under different political systems; for example, comparing social media platforms in the East and the West to see the similarities and differences in the effectiveness of using algorithmic censorship to suppress misinformation, especially pseudoscientific content with medical terminology. It is also possible to combine useful technologies in international research with governance experience in domestic research to develop a set of methods and tools that combine machine learning and social network analysis. In addition, observe the differences in the roles played by medical practitioners such as doctors and nurses in information dissemination in different countries and develop a set of scientifically based and universally applicable guidelines for professional social media behavior in different countries.

Second, we should pay attention to the changes in the information environment in the later stage of the epidemic. Most of the current research focuses on the period from 2020 to 2021 when the epidemic is serious, but some aspects are ignored: everyone’s scientific understanding of the “long-term COVID-19” is constantly updated, but the public’s understanding is not up to date, and the gap between them is changing; what long-term impact will the habit of frequent exposure to information during the epidemic have on other public health issues such as vaccination and antibiotic resistance; the structural changes of cross-border information dissemination networks, especially the transformation of the role of overseas accounts in new hot events. This type of long-term tracking research can refer to international long-term tracking methods for mental health and domestic analysis of public opinion fluctuations.

### Contribution and Novelty

Systematically carry out comparative bibliometric analysis of Chinese and Western information epidemic research: This research innovatively uses WOS and CNKI databases as samples and systematically compares the differences between China and the West in the information epidemic research of COVID-19 through bibliometric methods (such as CiteSpace visual analysis), filling the gap in the existing research that lacks cross-country and cross-database system comparison.

Clearly revealing the core differences and commonalities between Chinese and Western research: The study found that international research focuses on machine learning, social media analysis, and the impact of information epidemics on mental health, while Chinese research focuses on information governance, public opinion guidance, and information dissemination mechanisms. At the same time, it clarifies the commonalities between the two in the core goal of curbing the spread of misinformation, providing specific entry points for subsequent cross-cultural cooperation.

Pointing out the shortcomings of research cooperation and emphasizing the necessity of global collaboration: Through the analysis of the collaboration network between authors and institutions, the current situation of sparse collaboration between authors and institutions in Chinese and foreign research is revealed. The importance of establishing cross-border and interdisciplinary cooperation mechanisms is emphasized, providing a basis for building a globally unified information epidemic response system.

Provide practical and theoretical guidance for policy formulation and future research: Based on research differences, propose the possibility of integrating technological means and governance experience, provide reference for public health departments to formulate information management strategies, and point out directions for cross-cultural comparison and long-term information ecology tracking for subsequent research.

### Conclusions

As the world continues to confront the challenges of COVID-19, the concurrent information epidemic presents a complex issue. This paper provides a comprehensive analysis of five categories of sample data: publication trends, authorship, institutional affiliations, highly cited literature, and keyword topic clusters. Through this analysis, we have examined the evolution of the information epidemic and its multifaceted societal impacts, delineating its contours as perceived by scholars across different nations, institutions, and research orientations. This synthesis highlights research trajectories, differences in domestic and international approaches, and suggests strategies for addressing the information epidemic. Additionally, given the global impact of infodemics, integrating knowledge from various disciplines and regions is essential for fostering more effective responses.

## Abbreviations

CNKI: China National Knowledge Infrastructure
WOS: Web of Science
